# Supplementary material for: High-resolution micro-epidemiology of parasite spatial and temporal dynamics in a high malaria transmission setting in Kenya
Source: Nat Commun. 2019 Dec 9;10:5615. doi: 10.1038/s41467-019-13578-4 (PMC6901486; doi:10.1038/s41467-019-13578-4)
Supplement: Supplementary file 1 — Supplementary Information [file 41467_2019_13578_MOESM1_ESM.pdf]

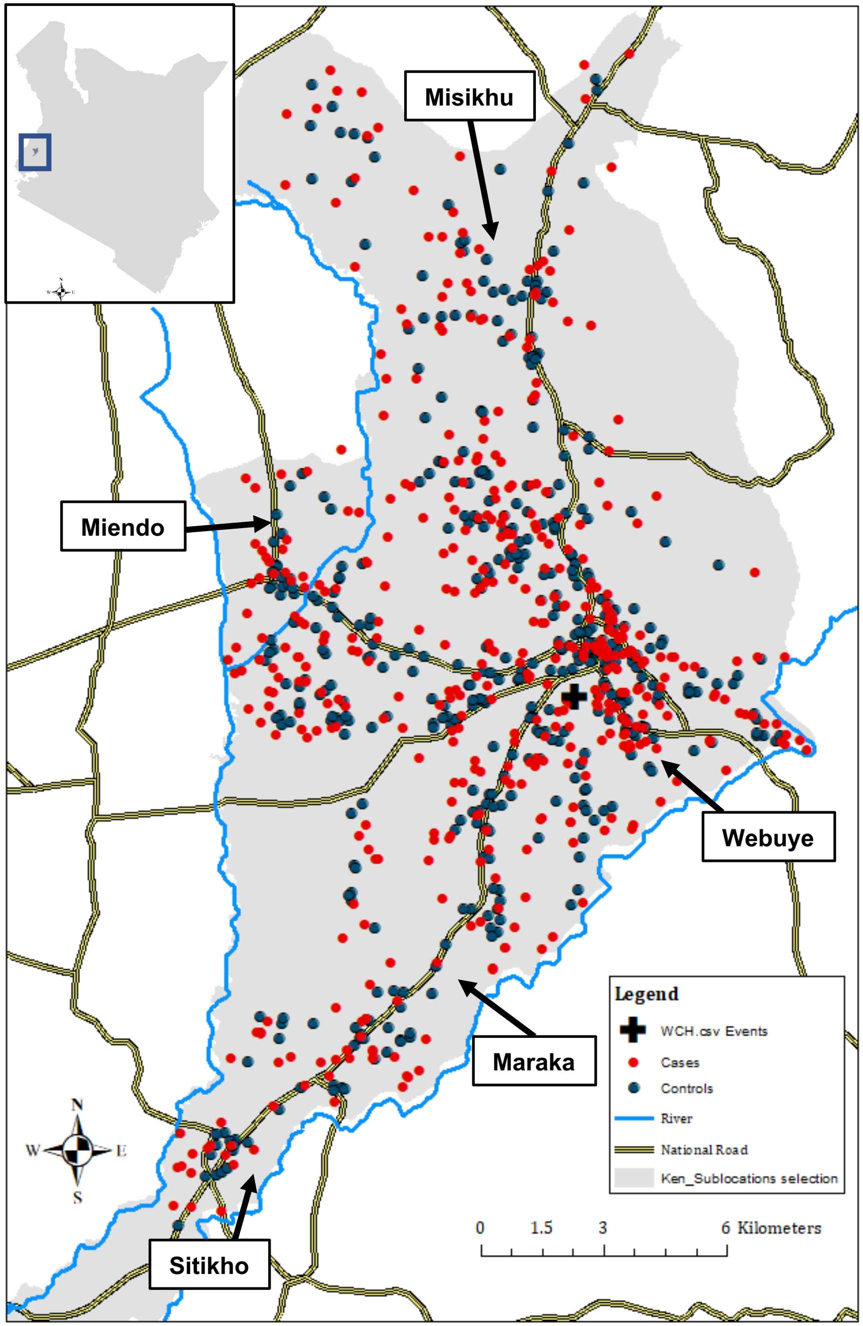

Supplementary Fig. 1 Map of study area in Bungoma county, western Kenya. Black cross indicates Webuye County Hospital (WCH), and gray outline denotes 10 administrative locations adjacent to the hospital. The 5 locations with the highest case incidence are labelled. The geographic location of the households of case and control children are indicated as red and blue dots, respectively. Blue denotes rivers, and yellow national roads. Visualization created using ArcGIS, version 10.7.

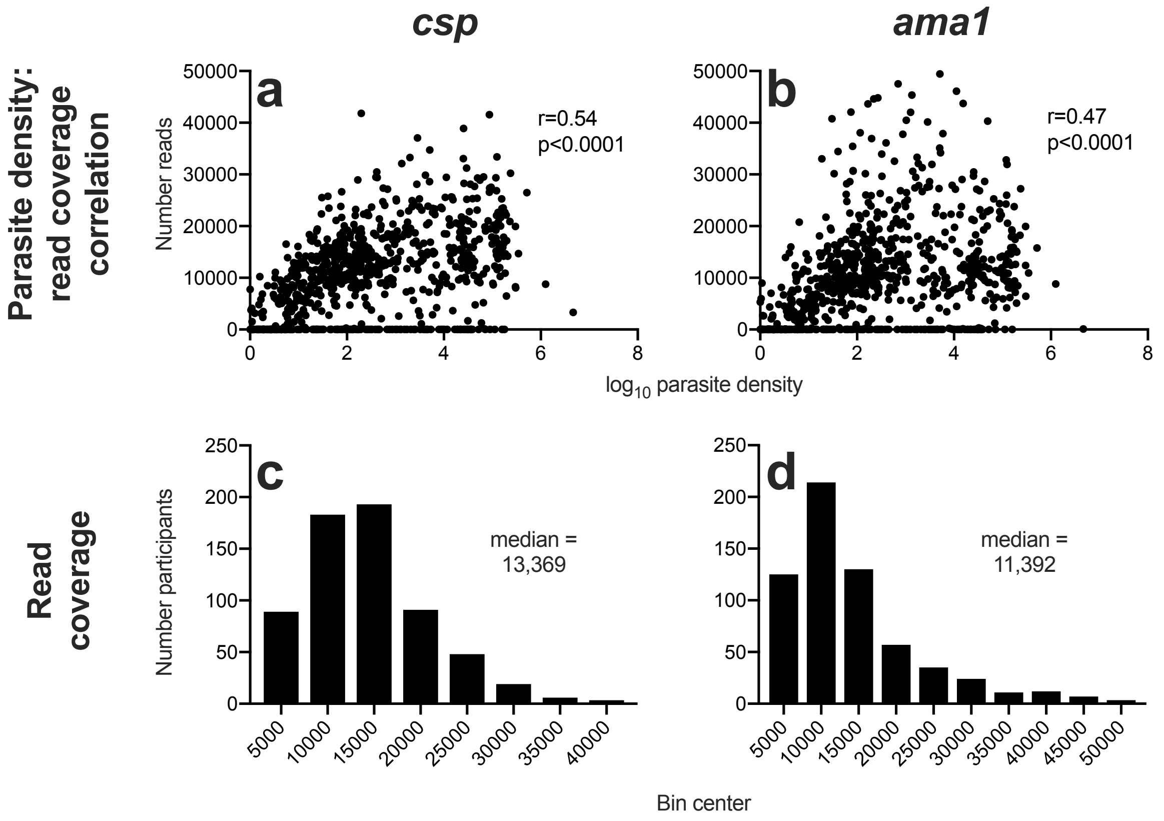

Supplementary Fig. 2 Parasite density is well correlated with read coverage. **a,b** Log_{10} parasite density for each sample is well correlated (Spearman rank test) with read coverage at *csp* (**a**) and *ama1* (**b**) loci. **c,d** Read coverage histograms for *csp* (**c**) and *ama1* (**d**) amplicons, indicating a median read coverage of 13,369 for the *csp* locus and 11,392 for the *ama1* locus.

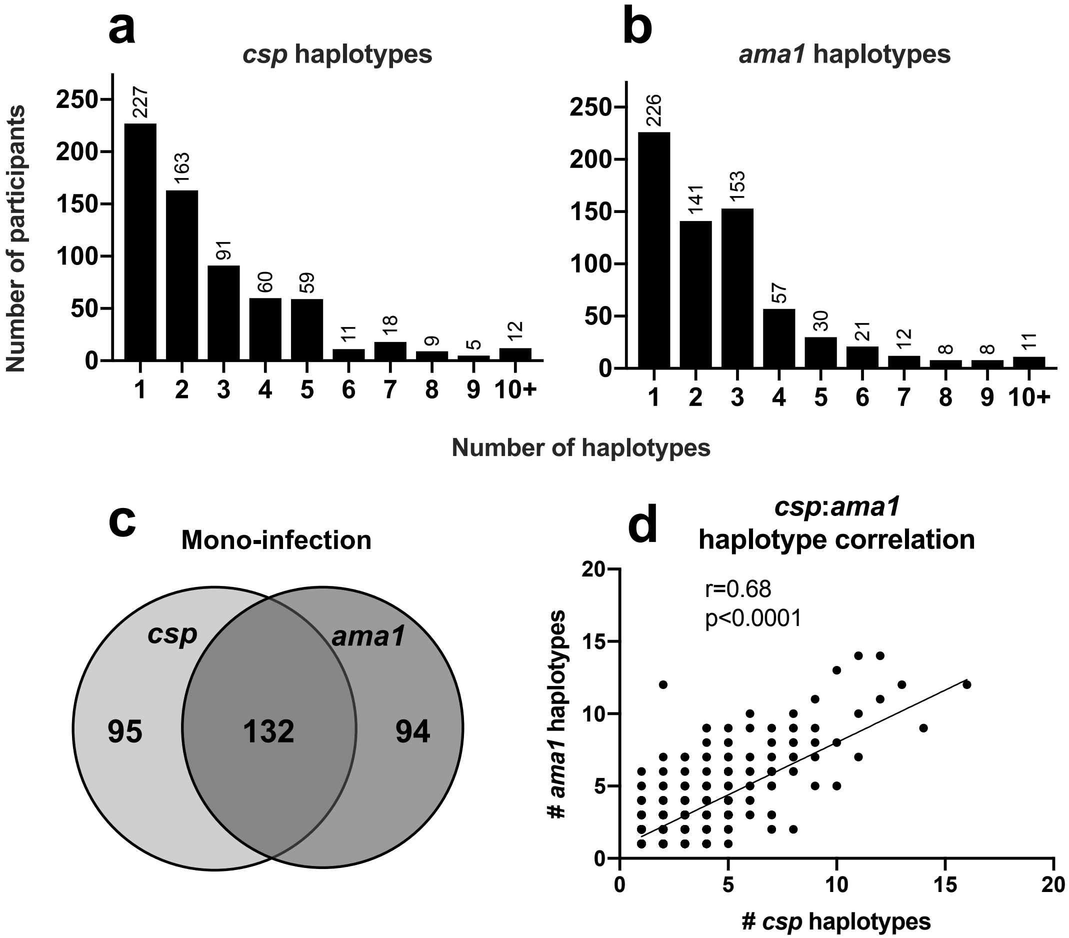

Supplementary Fig. 3 Multigenomic infections detected in majority of study participants. **a,b** Histogram denoting the number of *csp* (a) and *ama1* (a) haplotypes identified in study participants. (C) Majority of samples with apparent *csp* mono-infection also have *ama1* mono-infection and vice versa. (D) Number of haplotypes identified at *csp* and *ama1* loci are well correlated.

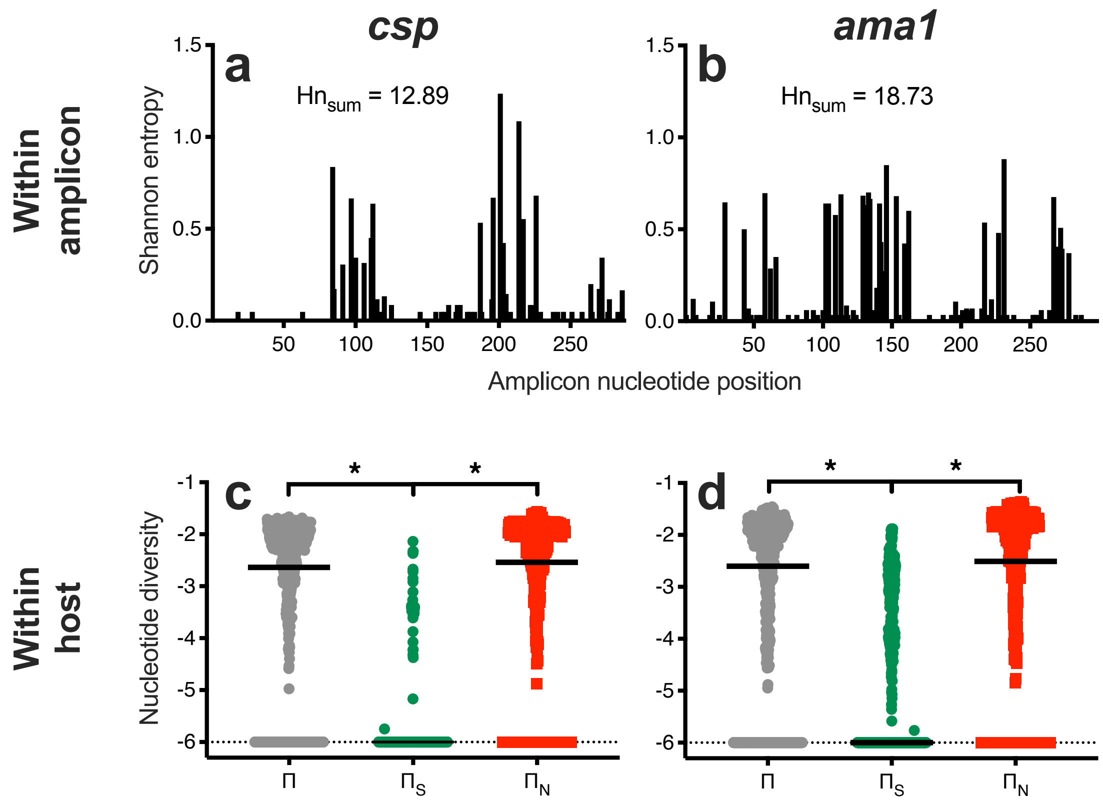

Supplementary Fig. 4 Nucleotide diversity within amplicons and within hosts. **a,b** Shannon entropy scores at each position of the *csp* (**a**) and *ama1* (**b**) amplicons indicate that sequence diversity is restricted to 3 discrete regions within the csp amplicon though more evenly distributed in the *ama1* amplicon. Furthermore, the *ama1* amplicon has enhanced diversity overall with a total entropy (Hn_{sum}) of 18.73 compared with 12.89 for *csp.* **c,d** Intrahost nucletotide diversity (Π) is predominantly nonsynonymous (Π_N) rather than synonymous (Π_S) for both *csp* (**c**) and *ama1* (**d**) amplicons. * $p<0.001$, Friedman test + posthoc Wilcoxon Signed-Rank test.

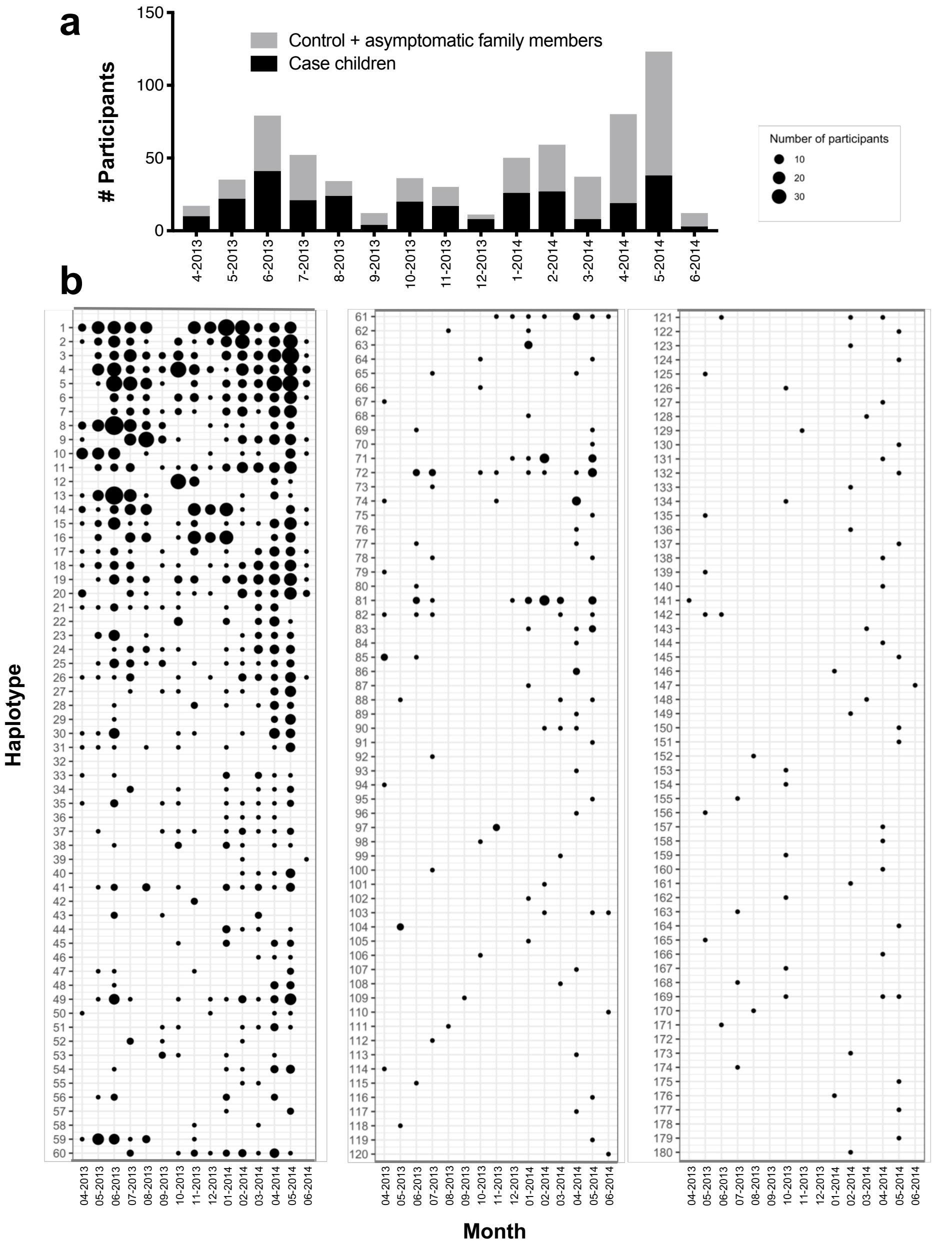

Supplementary Fig. 5 *ama1* unique haplotype prevalence by month. **a** Total number of study participants with *ama1* haplotypes by month. Black denotes case children, and gray indicates both control and case household members. **b** Monthly prevalence of 180 unique *ama1* haplotypes, sorted by overall prevalence. Size of circle indicates number of study participants sharing a particular haplotype in a given month.

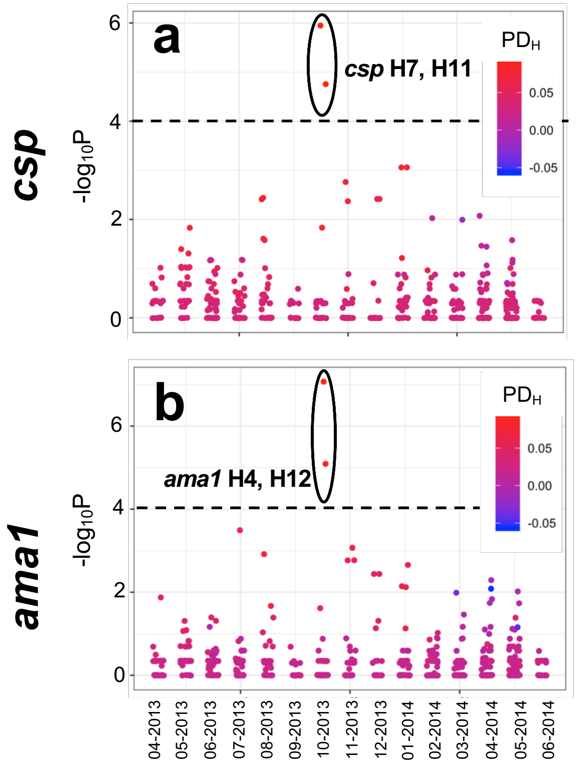

Supplementary Fig. 6 No consistent haplotype bias by age **a,b** The haplotype prevalence difference (PD_H) between young children ($\leq$ 5y; n=296 for *csp* and 300 for *ama1*) and older children/adults (>5y; n=357 for *csp* and 365 for *ama1*) during each month was calculated for *csp* (**a**) and *ama1* (**b**). Color indicates PD_H, with red identifying haplotypes more common in $\leq$ 5y population and blue more common in >5y population. The y-axis indicates the Fisher's Exact test $-\log_{10}$(p-value) for haplotype prevalence in $\leq$ 5y vs. >5y groups, while the dotted line denotes the Bonferroni-corrected threshold for statistical significance.

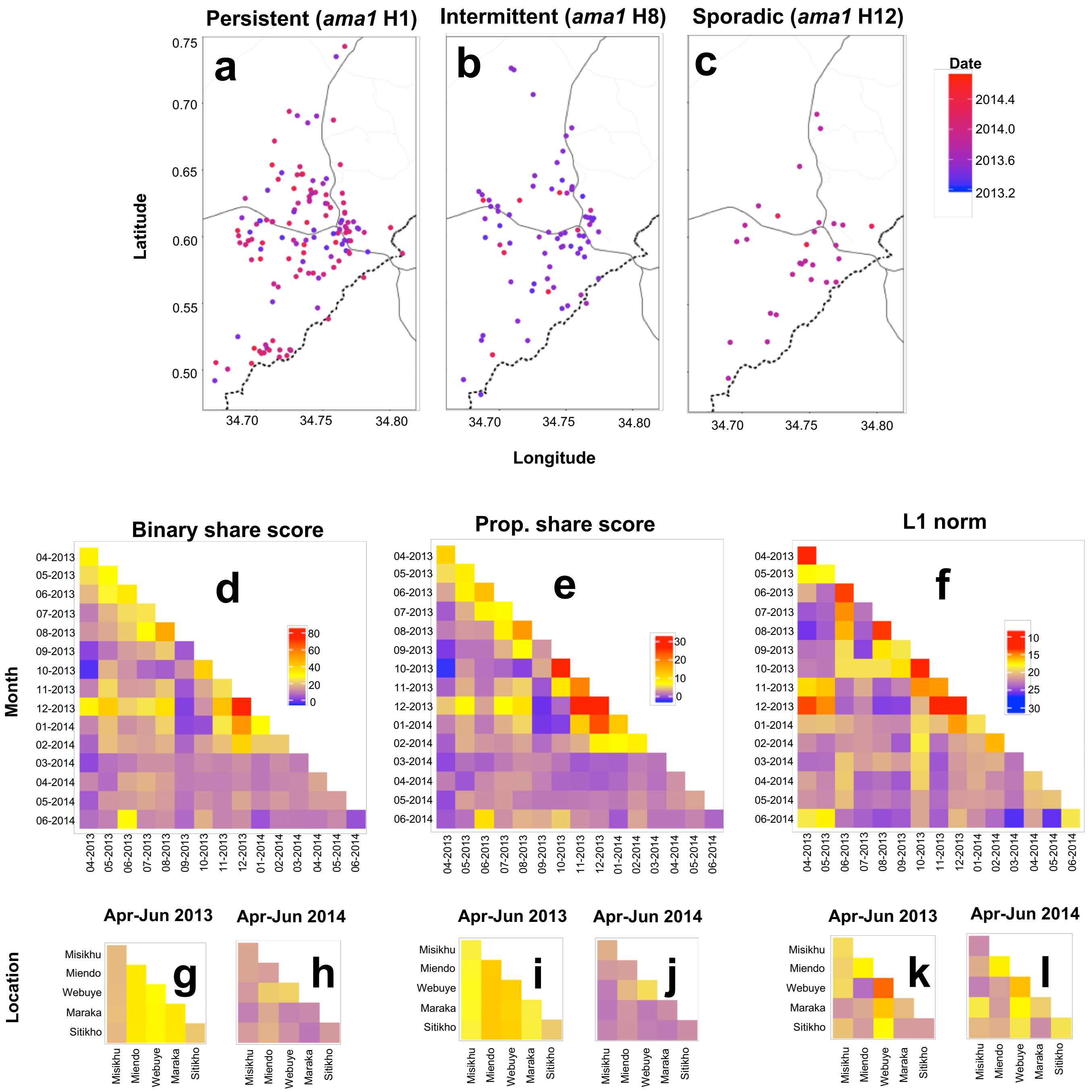

Persistent (ama1 H1)
Intermittent (ama1 H8)
Sporadic (ama1 H12)
a
b
c
Latitude
0.75
0.70
0.65
0.60
0.55
0.50
34.70
34.75
34.80
Longitude
Date
2014.4
2014.0
2013.6
2013.2
Binary share score
Prop. share score
L1 norm
d
e
f
Month
04-2013
05-2013
06-2013
07-2013
08-2013
09-2013
10-2013
11-2013
12-2013
01-2014
02-2014
03-2014
04-2014
05-2014
06-2014
80
60
40
20
0
30
20
10
0
10
15
20
25
30
Apr-Jun 2013
Apr-Jun 2014
Location
Misikhu
Miendo
Webuye
Maraka
Sitikho
g
h
i
j
k
l

Supplementary Fig. 7. Genetic similarity of *ama1* haplotypes is structured by time more than space. **a-c** Location of study participants with ‘persistent’ haplotype *ama1* H1 (**a**), ‘intermittent’ haplotype *ama1* H8 (**b**), and ‘sporadic’ haplotype *ama1* H12 (**c**). Blue color indicates the beginning (April 2013) and red the end (June 2014) of the study period, with the date denoting fractional years in decimal notation. **d-f** Temporal comparison heat maps of mean binary haplotype sharing (**d**), proportional haplotype sharing (**e**), and L1 norm genetic distance (**f**) calculated between months of study enrollment. **g-l** Spatial comparison heat maps of binary haplotype sharing (**g,h**), proportional haplotype sharing (**i,j**), and L1 norm genetic distance (**k,l**) calculated for a distinct temporal window (**g,i,k**: April-June 2013; **h,j,l**: April-June 2014) for the 5 most represented administrative locations (see map in Figure S1), which are arranged geographically from north to south. For **d-l**, blue denotes the minimum for each genetic similarity index, red the maximum, and yellow the midpoint.

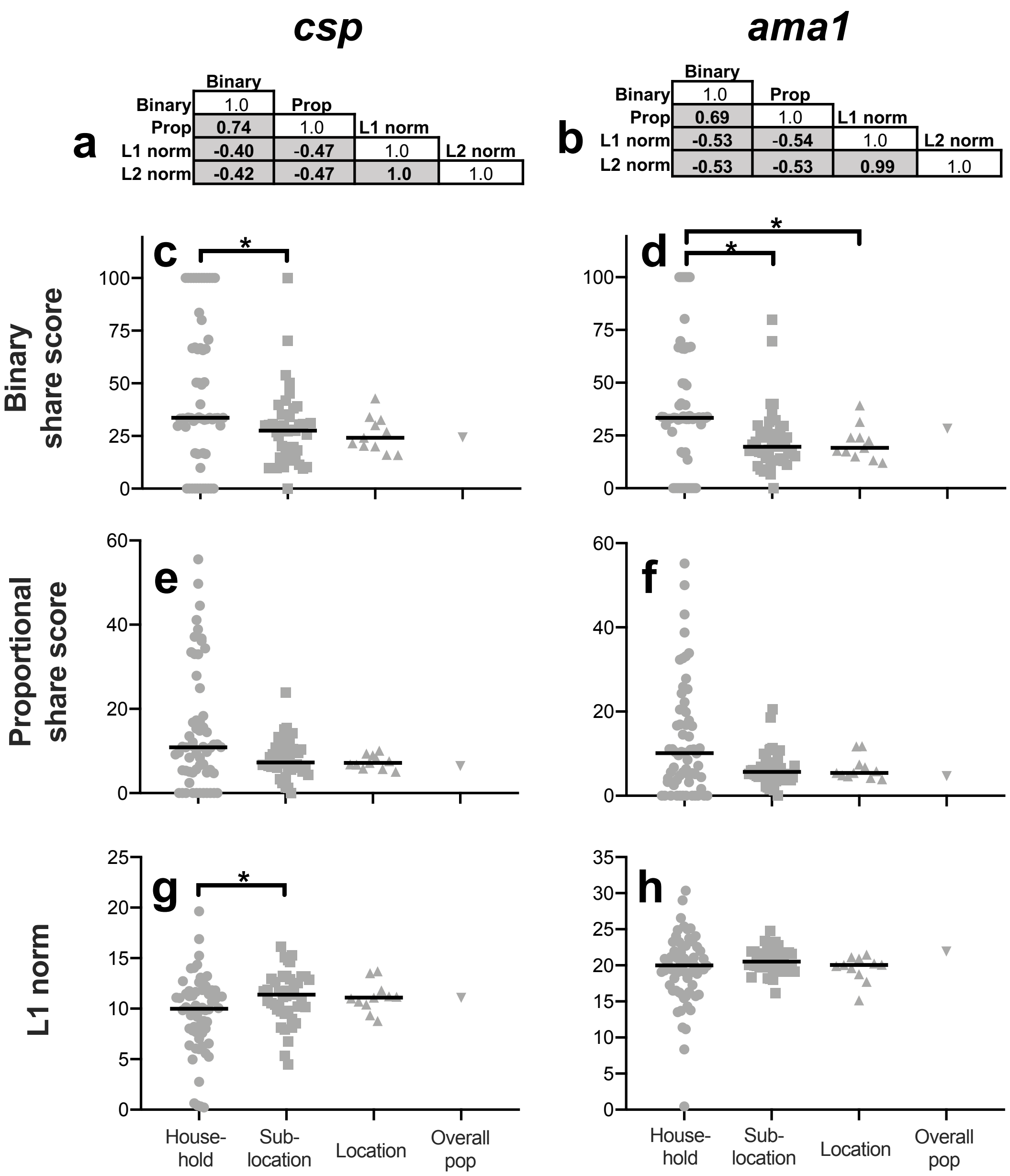

Supplementary Fig. 8 Binary and proportional share scores are directly correlated, and inversely with L1/L2 norm metrics. **a,b** Correlation matrix (Spearman rank test) for binary share score, proportional share score (prop), L1 norm, and L2 norm calculated for *csp* (**a**) and *ama1* (**b**) loci. **c-h** Mean *csp* /*ama1* binary share score (**c,d**), proportional share score (**e,f**), and L1 norm (**g,h**) calculated for combination of individuals comprising each distinct household with 3+ members, sublocation, location, and for the overall population. *p<0.05, Mann-Whitney U test.

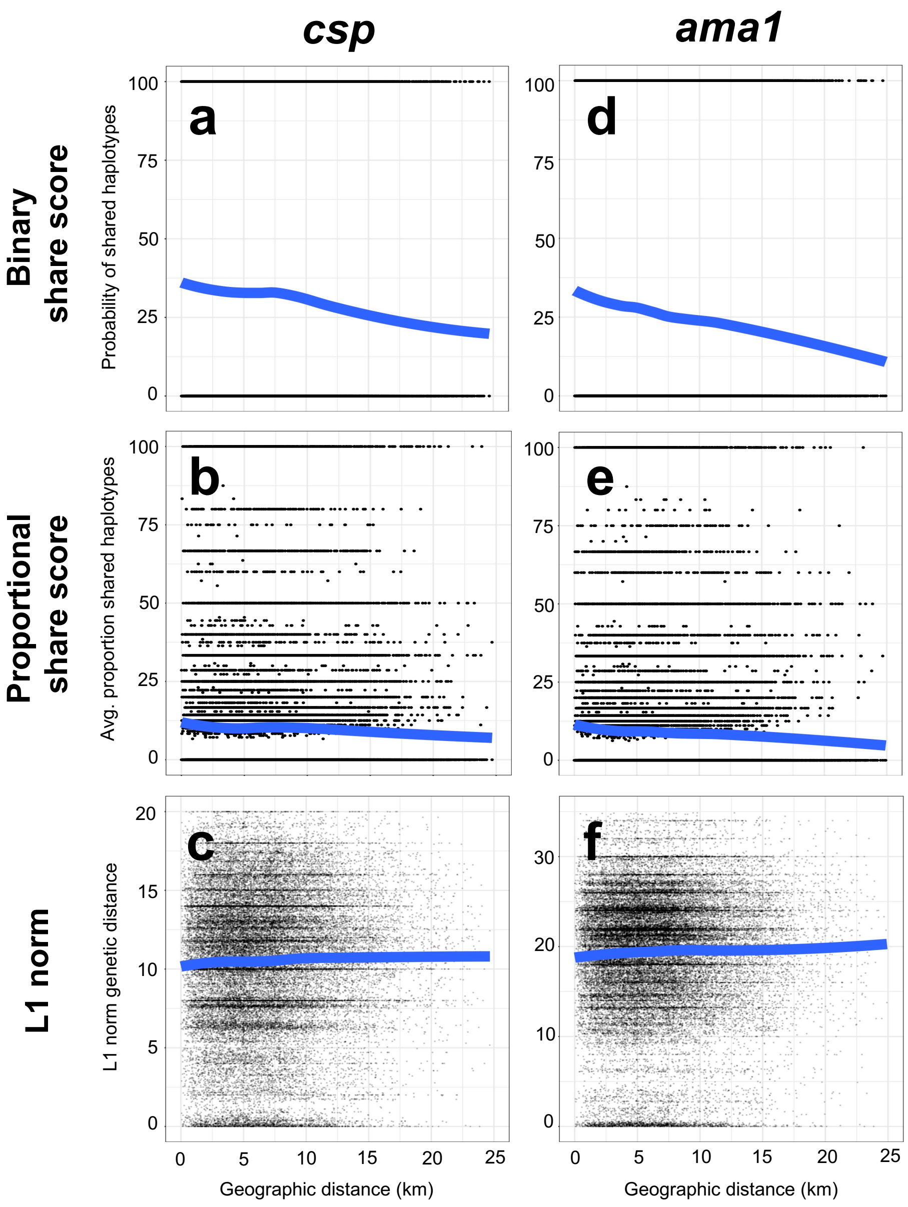

Supplementary Fig. 9 No clear enhanced genetic similarity of infections among geographically-proximal symptomatic children. Genetic similarity metrics were computed for all possible pairings of case children for *csp* (n=273) and *ama1* (n=288) haplotypes. **a-c** *csp* haplotype binary sharing (**a**), proportional sharing (**b**) and L1 norm (**c**) metrics for all CC pairwise comparisons is plotted against geographic distance for CC. **d-f** *ama1* haplotype binary sharing (**d**), proportional sharing (**e**) and L1 norm (**f**) metrics for all CC pairings is plotted against geographic distance for CC. Blue lines indicate the locally-estimated scatterplot smoothing (LOESS) regression fit of data.

Supplementary Table 1 Statistical comparison of study participants successfully/unsuccessfully assigned CSP and AMA haplotypes

		**Successful haplotype assignment**	**Unsuccessful haplotype assignment**	**P-value**	**Test**
	Median $\log_{10}$ PD (range)	2.36 (-0.51 – 6.67)	0.71 (-0.86 – 5.85)	**p<0.001**	Mann-Whitney U Test
csp	Median age (range)	6 (0.08 – 82)	6 (0.08 – 73.5)	p=0.70	Mann-Whitney U Test
	Case child percentage	44.60%	31.20%	**p<0.001**	Fisher's Exact Test
	Median $\log_{10}$ PD (range)	2.31 (-0.86 – 6.67)	0.74 (-0.66 – 5.48)	**p<0.001**	Mann-Whitney U Test
ama1	Median age (range)	6 (0.08 – 82)	6 (0.08 – 73.5)	p=0.75	Mann-Whitney U Test
	Case child percentage	43.20%	31.20%	**p<0.001**	Fisher's Exact Test

Supplementary Table 2 Comparison of genetic similarity metrics within-month

		**Binary sharing**	**Proportional sharing**	**L1-norm**
	Within-month median (n=15)	36.17	13.61	8.51
csp	Different month median (n=105)	23.28	5.91	11.36
	P-value (Mann-Whitney U test)	**0.029**	<**0.001**	**0.013**
	Within-month median (n=15)	26.63	9.60	18.07
ama1	Different month median (n=105)	15.80	3.82	21.68
	P-value (Mann-Whitney U test)	**0.004**	**<0.001**	**<0.001**

Supplementary Table 3 Comparison of genetic similarity metrics within-location

		Binary sharing		Proportional sharing		L1-norm	
		Apr-Jun 2013	Apr-Jun 2014	Apr-Jun 2013	Apr-Jun 2014	Apr-Jun 2013	Apr-Jun 2014
csp	Within-location median (n=5)	50.34	26.43	7.64	13.94	7.47	7.87
	Different location median (n=10)	53.69	18.34	4.12	15.11	8.96	8.96
	P-value (Mann-Whitney U)	0.594	0.076	**0.002**	0.371	0.240	0.165
ama1	Within-location median (n=5)	30.52	17.45	7.43	4.70	19.23	18.95
	Different location median (n=10)	31.57	16.87	9.95	3.69	20.52	20.79
	P-value (Mann-Whitney U)	1.000	0.514	0.207	0.075	0.207	0.254

Supplementary Table 4 Round 1 primers

Name	Sequence*
PfcspOH-F	TCGTCGGCAGCGTC**AGATGTGTATAAGAGACAG**TTAAGGAACAAGAAGGATAATACCA
PfcspOH-R	GTCTCGTGGGCTCGG**AGATGTGTATAAGAGACAG**AAATGACCCAAACCGAAATG
PfamaOH-F	TCGTCGGCAGCGTC**AGATGTGTATAAGAGACAGT**CAGGGAAATGTCCAGTATTTG
PfamaOH-R	GTCTCGTGGGCTCGG**AGATGTGTATAAGAGACAG**GGACCATTATTTTCTTGAGCTG

*Non-bolded, non-shaded sequence is complementary to the target sequence in the corresponding gene. Bolded sequence is the overhang sequence that is intentionally included in the PCR products in order to enable downstream MiSeq sequencing (Illumina Miseq Library prep guide). Shadowed is the portion of this overhang sequence that will be targeted for binding by the second-round primers.

Supplementary Table 5 Round 2 MiSeq primers

Name	Index	Sequence*
P5MiSeqi1	AACCAAGG	**AATGATACGGCGACCACCGAGATCTACAC**AACCAAGGTCGTCGGCAGCGTC
P5MiSeqi2	AAGGTACG	**AATGATACGGCGACCACCGAGATCTACAC**AAGGTACGTCGTCGGCAGCGTC
P5MiSeqi3	ACCTACCT	**AATGATACGGCGACCACCGAGATCTACAC**ACCTACCTTCGTCGGCAGCGTC
P5MiSeqi4	ACGTGTTG	**AATGATACGGCGACCACCGAGATCTACAC**ACGTGTTGTCGTCGGCAGCGTC
P5MiSeqi5	ACTGGACT	**AATGATACGGCGACCACCGAGATCTACAC**ACTGGACTTCGTCGGCAGCGTC
P5MiSeqi6	AGAGACTG	**AATGATACGGCGACCACCGAGATCTACAC**AGAGACTGTCGTCGGCAGCGTC
P5MiSeqi7	AGTCGACT	**AATGATACGGCGACCACCGAGATCTACAC**AGTCGACTTCGTCGGCAGCGTC
P5MiSeqi8	ATATGCCG	**AATGATACGGCGACCACCGAGATCTACAC**ATATGCCGTCGTCGGCAGCGTC
P5MiSeqi9	CAACCATG	**AATGATACGGCGACCACCGAGATCTACAC**CAACCATGTCGTCGGCAGCGTC
P5MiSeqi10	CACAGTGT	**AATGATACGGCGACCACCGAGATCTACAC**CACAGTGTTCGTCGGCAGCGTC
P5MiSeqi11	CAGAAGTG	**AATGATACGGCGACCACCGAGATCTACAC**CAGAAGTGTCGTCGGCAGCGTC
P5MiSeqi12	CAGTGACT	**AATGATACGGCGACCACCGAGATCTACAC**CAGTGACTTCGTCGGCAGCGTC
P5MiSeqi13	CATGTGGT	**AATGATACGGCGACCACCGAGATCTACAC**CATGTGGTTCGTCGGCAGCGTC
P5MiSeqi14	CTTCGAAG	**AATGATACGGCGACCACCGAGATCTACAC**CTTCGAAGTCGTCGGCAGCGTC
P5MiSeqi15	CATCGATG	**AATGATACGGCGACCACCGAGATCTACAC**CATCGATGTCGTCGGCAGCGTC
P5MiSeqi16	CGTAGGAA	**AATGATACGGCGACCACCGAGATCTACAC**CGTAGGAATCGTCGGCAGCGTC
P5MiSeqi17	CTAGTGGT	**AATGATACGGCGACCACCGAGATCTACAC**CTAGTGGTTCGTCGGCAGCGTC
P5MiSeqi18	CTCTGACT	**AATGATACGGCGACCACCGAGATCTACAC**CTCTGACTTCGTCGGCAGCGTC
P5MiSeqi19	CTGTGAGT	**AATGATACGGCGACCACCGAGATCTACAC**CTGTGAGTTCGTCGGCAGCGTC
P5MiSeqi20	GAACCTTG	**AATGATACGGCGACCACCGAGATCTACAC**GAACCTTGTCGTCGGCAGCGTC
P5MiSeqi21	GACATCTG	**AATGATACGGCGACCACCGAGATCTACAC**GACATCTGTCGTCGGCAGCGTC
P5MiSeqi22	GAGAGACT	**AATGATACGGCGACCACCGAGATCTACAC**GAGAGACTTCGTCGGCAGCGTC
P5MiSeqi23	GATCGAAG	**AATGATACGGCGACCACCGAGATCTACAC**GATCGAAGTCGTCGGCAGCGTC
P5MiSeqi24	GCAATAGG	**AATGATACGGCGACCACCGAGATCTACAC**GCAATAGGTCGTCGGCAGCGTC
P7MiSeqi25	GCTACCTT	**CAAGCAGAAGACGGCATACGAGAT**GCTACCTTGTCTCGTGGGCTCGG
P7MiSeqi26	GACACTGT	**CAAGCAGAAGACGGCATACGAGAT**GACACTGTGTCTCGTGGGCTCGG
P7MiSeqi27	GGTTCCTT	**CAAGCAGAAGACGGCATACGAGAT**GGTTCCTTGTCTCGTGGGCTCGG
P7MiSeqi28	GTCATCAG	**CAAGCAGAAGACGGCATACGAGA**TGTCATCAGGTCTCGTGGGCTCGG
P7MiSeqi29	GTGAGTCT	**CAAGCAGAAGACGGCATACGAGA**TGTGAGTCTGTCTCGTGGGCTCGG
P7MiSeqi30	GTTCGATG	**CAAGCAGAAGACGGCATACGAGA**TGTTCGATGGTCTCGTGGGCTCGG
P7MiSeqi31	TACGATCG	**CAAGCAGAAGACGGCATACGAGA**TTACGATCGGTCTCGTGGGCTCGG
P7MiSeqi32	TCACTCTG	**CAAGCAGAAGACGGCATACGAGA**TTCACTCTGGTCTCGTGGGCTCGG
P7MiSeqi33	TCTCCAGT	**CAAGCAGAAGACGGCATACGAGA**TTCTCCAGTGTCTCGTGGGCTCGG
P7MiSeqi34	TGACTCAG	**CAAGCAGAAGACGGCATACGAGA**TTGACTCAGGTCTCGTGGGCTCGG
P7MiSeqi35	TGGTTCCT	**CAAGCAGAAGACGGCATACGAGA**TTGGTTCCTGTCTCGTGGGCTCGG
P7MiSeqi36	TGTGACTG	**CAAGCAGAAGACGGCATACGAGA**TTGTGACTGGTCTCGTGGGCTCGG
P7MiSeqi37	TGTGACTG	**CAAGCAGAAGACGGCATACGAGA**TTGTGACTGGTCTCGTGGGCTCGG
P7MiSeqi38	ACACGACT	**CAAGCAGAAGACGGCATACGAGA**TACACGACTGTCTCGTGGGCTCGG
P7MiSeqi39	ACTGTCAG	**CAAGCAGAAGACGGCATACGAGA**TACTGTCAGGTCTCGTGGGCTCGG
P7MiSeqi40	AGTCTCAG	**CAAGCAGAAGACGGCATACGAGA**TAGTCTCAGGTCTCGTGGGCTCGG
P7MiSeqi41	ATCCAACG	**CAAGCAGAAGACGGCATACGAGA**TATCCAACGGTCTCGTGGGCTCGG
P7MiSeqi42	CAACCTAG	**CAAGCAGAAGACGGCATACGAGA**TCAACCTAGGTCTCGTGGGCTCGG
P7MiSeqi43	CACATCAG	**CAAGCAGAAGACGGCATACGAGA**TCACATCAGGTCTCGTGGGCTCGG
P7MiSeqi44	CAGACTGT	**CAAGCAGAAGACGGCATACGAGA**TCAGACTGTGTCTCGTGGGCTCGG
P7MiSeqi45	GTCTACAG	**CAAGCAGAAGACGGCATACGAGA**TGTCTACAGGTCTCGTGGGCTCGG
P7MiSeqi46	GTGATGAG	**CAAGCAGAAGACGGCATACGAGA**TGTGATGAGGTCTCGTGGGCTCGG
P7MiSeqi47	GTTGGTTG	**CAAGCAGAAGACGGCATACGAGA**TGTTGGTTGGTCTCGTGGGCTCGG
P7MiSeqi48	TACGTACG	**CAAGCAGAAGACGGCATACGAGA**TTACGTACGGTCTCGTGGGCTCGG

*Bolded sequence is either the P5 or the P7 adaptor. Shaded sequence is the sequence that is complementary to the overhang sequence present in the 1st round products.
