# Supplementary material for: High-resolution micro-epidemiology of parasite spatial and temporal dynamics in a high malaria transmission setting in Kenya
Source: Nat Commun. 2019 Dec 9;10:5615. doi: 10.1038/s41467-019-13578-4 (PMC6901486; doi:10.1038/s41467-019-13578-4)
Supplement: Supplementary file 3 — Reporting Summary [file 41467_2019_13578_MOESM3_ESM.pdf]

nature research

Corresponding author(s): *Double-blind peer review submissions: write DBPR and your manuscript number here instead of author names.*

Last updated by author(s): 10/19/2019

Reporting Summary

Nature Research wishes to improve the reproducibility of the work that we publish. This form provides structure for consistency and transparency in reporting. For further information on Nature Research policies, seeAuthors & Referees and theEditorial Policy Checklist .

Statistics

For all statistical analyses, confirm that the following items are present in the figure legend, table legend, main text, or Methods section.

n/a	Confirmed	
☐	☒	The exact sample size (*n*) for each experimental group/condition, given as a discrete number and unit of measurement
☐	☒	A statement on whether measurements were taken from distinct samples or whether the same sample was measured repeatedly
☐	☒	The statistical test(s) used AND whether they are one- or two-sided<br>*Only common tests should be described solely by name; describe more complex techniques in the Methods section.*
☒	☐	A description of all covariates tested
☒	☐	A description of any assumptions or corrections, such as tests of normality and adjustment for multiple comparisons
☐	☒	A full description of the statistical parameters including central tendency (e.g. means) or other basic estimates (e.g. regression coefficient) AND variation (e.g. standard deviation) or associated estimates of uncertainty (e.g. confidence intervals)
☐	☒	For null hypothesis testing, the test statistic (e.g. *F*, *t*, *r*) with confidence intervals, effect sizes, degrees of freedom and *P* value noted<br>*Give P values as exact values whenever suitable.*
☒	☐	For Bayesian analysis, information on the choice of priors and Markov chain Monte Carlo settings
☒	☐	For hierarchical and complex designs, identification of the appropriate level for tests and full reporting of outcomes
☒	☐	Estimates of effect sizes (e.g. Cohen's *d*, Pearson's *r*), indicating how they were calculated

Our web collection on statistics for biologists contains articles on many of the points above.

Software and code

Policy information about availability of computer code

Data collection	No software was used
Data analysis	All analyses were completed in R version 3.5.1. Source data and code for analyses is available for download from GitHub: https://github.com/codynelson08/MESAseq_compiled_code.

For manuscripts utilizing custom algorithms or software that are central to the research but not yet described in published literature, software must be made available to editors/reviewers. We strongly encourage code deposition in a community repository (e.g. GitHub). See the Nature Research guidelines for submitting code & software for further information.

Data

Policy information about availability of data

All manuscripts must include a data availability statement. This statement should provide the following information, where applicable:

- Accession codes, unique identifiers, or web links for publicly available datasets
- A list of figures that have associated raw data
- A description of any restrictions on data availability

Haplotype sequences are available in NCBI GenBank under accessioning numbers MK933826 – MK933945 (csp) and MK933946 – MK934125 (ama1). All analyses were completed in R version 3.5.1. Source data and code for analyses is available for download from GitHub: https://github.com/codynelson08/MESAseq_compiled_code. Raw analysis results available upon request.

Field-specific reporting

Please select the one below that is the best fit for your research. If you are not sure, read the appropriate sections before making your selection.

Life sciences study design

All studies must disclose on these points even when the disclosure is negative.

Sample size	No sample size calculation was performed. Rather, sample size was dictated by the number of case children with RDT+ malaria (+ matched control children, + household members of both groups) who could be enrolled at Webuye County Hospital within the 15 month study time frame.
Data exclusions	See figure 1. Only RDT+ samples were selected for sequencing. A subset of those individuals (~30%) failed quality control, PCR amplification, or were not assigned haplotypes (likely due to low parasite density). Furthermore, a small number of samples were omitted due to inconsistencies or erroneous sample tracking/identification.
Replication	Samples were not sequenced/analyzed in duplicate. However, we did sequence all samples at two unlinked gene targets (csp and ama1) and observed concordant findings throughout all analyses.
Randomization	Samples were assigned unique IDs and sorted randomly into plates for DNA extraction, PCR amplification, and sequencing. Only after haplotype calling were samples matched with clinical/demographic data (using randomized IDs) for subsequent analysis.
Blinding	Research staff carrying out the DNA extraction, PCR amplification, and sequencing were completely blinded to sample identity.

Reporting for specific materials, systems and methods

We require information from authors about some types of materials, experimental systems and methods used in many studies. Here, indicate whether each material, system or method listed is relevant to your study. If you are not sure if a list item applies to your research, read the appropriate section before selecting a response.

Materials & experimental systems

n/a	Involved in the study	
☒	☐	Antibodies
☒	☐	Eukaryotic cell lines
☒	☐	Palaeontology
☒	☐	Animals and other organisms
☐	☒	Human research participants
☒	☐	Clinical data

Methods

n/a	Involved in the study	
☒	☐	ChIP-seq
☒	☐	Flow cytometry
☒	☐	MRI-based neuroimaging

Human research participants

Policy information about studies involving human research participants

Population characteristics	Children (<10 years old) admitted to the Webuye County Hospital with a diagnosis of malaria (confirmed by SD Bioline Pf HRP2 RDT) who resided within the six administrative sublocations immediately surrounding the hospital were eligible for enrollment as case children (CC). Household members of CC were tested by RDT and provided a dried blood spot (DBS) within 1-7 days of enrolling the CC. At that time, CC were matched by age and village to an RDT-negative control child and all of the members of the control child's household were similarly tested and provided a DBS.
Recruitment	See above. Since we sought to examine temporal/spatial structuring of parasite populations, potential biases arise from the lack of longitudinal sampling and the fact that each child (and surrounding household) represents a single point in both time and space. It is therefore difficult to parse out temporal vs. spatial aggregation of genetic similarity.
Ethics oversight	The study protocol and consent procedures were reviewed and approved by the Moi University Institutional Research and Ethics Committee (IREC/2013/13) and the Duke University Institutional Review Board (Pro00044098).

Note that full information on the approval of the study protocol must also be provided in the manuscript.
